# The elimination of miR-23a in heat-stressed cells promotes NOXA-induced cell death and is prevented by HSP70

**DOI:** 10.1038/cddis.2014.484

**Published:** 2014-11-27

**Authors:** R Roufayel, D S Johnston, D D Mosser

**Affiliations:** 1 Department of Molecular and Cellular Biology, University of Guelph, 50 Stone Road East, Guelph, Ontario, N1G 2W1, Canada

## Abstract

Protein-damaging stress stimulates cell destruction through apoptosis; however, non-lethal proteotoxic stress induces an adaptive response leading to the increased synthesis of heat shock proteins, which inhibit apoptosis. In this study, we sought to determine the mechanism responsible for the accumulation of the BH3-only protein NOXA in heat-stressed cells and its prevention by the heat shock protein HSP70. Analysis of transcript levels by RT-qPCR revealed that miR-23a levels decreased in heat-stressed cells and that this was correlated with an increased abundance of NOXA mRNA, which contains a miR-23a binding site in its 3′ untranslated region. Cells overexpressing HSP70 had higher levels of miR-23a, maintained these levels after heat shock and accumulated lower levels of NOXA mRNA and protein. The enhanced abundance of mir-23a in these HSP70-expressing cells is primarily due to its increased stability although higher levels of pri/pre-miR-23a expression, nuclear export and maturation were also contributing factors. Stable overexpression of miR-23a in the acute lymphoblastic T-cell line PEER resulted in reduced basal and heat-induced levels of NOXA mRNA and significantly inhibited heat-induced apoptosis. Additionally, stable overexpression of an shRNA targeting miR-23a in U937 lymphoma cells produced stable knockdown of miR-23a and resulted in increased NOXA mRNA and an increased sensitivity to heat-induced apoptosis. These results demonstrate the novel finding that hyperthermia affects the abundance of a microRNA that targets the expression of a pro-apoptotic protein and that HSP70 protects cells from heat-induced apoptosis by regulating the abundance of this microRNA. We speculate that the inhibition of miRNA transcription in heat-stressed cells could represent a general mechanism for apoptosis induction that is regulated by the molecular chaperone protein HSP70. Furthermore, we propose that HSP70 could be beneficial to tumor cells by helping to maintain the expression of oncogenic miRNAs under conditions of cellular stress.

Protein-damaging stress, such as exposure to elevated temperature, can activate a process of cellular destruction known as apoptosis. Exposure to hyperthermia also induces the synthesis of heat shock proteins including HSP70 (HSPA1A), which can protect cells from stress-induced apoptosis.^1, 2^ Cell death is regulated by pro- and anti-apoptotic members of the BCL2 family.^3, 4^ The pro-apoptotic members BAX and BAK oligomerize and form channels in the mitochondrial outer membrane of stressed cells permitting the release of pro-apoptotic factors that result in caspase activation leading to the proteolytic dismantling of the dying cell. Anti-apoptotic members of the BCL2 family, BCL2, MCL1, BCL-X_L_, BCL2A1 and BCL-W, prevent BAX/BAK oligomerization through direct physical interactions. The pro-apoptotic BH3-only (BCL2 homology domain 3) proteins, BIM, BAD, BID, NOXA (PMAIP1) and PUMA, ultimately control cell fate by interacting with the anti-apoptotic members and relieving their ability to suppress BAX/BAK oligomerization or, in the case of some (BIM, BID), by directly stimulating BAX/BAK activation. Although some BH3-only proteins can inhibit all anti-apoptotic BCL2 proteins, NOXA is only able to interact with MCL1 and A1.^5^ Both NOXA and MCL1 have short half-lives and therefore their abundance can be rapidly altered in stressed cells.^6, 7^ NOXA, which is bound to the mitochondrial outer membrane, binds cytosolic MCL1 leading to its phosphorylation, ubiquitination and proteasomal degradation.^8, 9^ This in turn frees BIM from MCL1 sequestration enabling BAX/BAK oligomerization.^10^

Apoptosis in heat-stressed cells occurs through BAX activation.^11, 12^ This is mediated in part by a NOXA-dependent depletion of MCL1 protein that is regulated by HSP70.^13^ Although NOXA protein levels initially drop after cells are exposed to hyperthermia, they subsequently increase to levels greater than that of non-stressed cells.^13^ In this study, we sought to investigate whether this increase in NOXA protein levels is regulated posttranscriptionally by miRNA-mediated suppression. As microRNAs are generally transcribed by RNA polymerase II, which is inhibited by hyperthermia,^14, 15^ we reasoned that the increased expression of NOXA in heat-stressed cells could be the result of decreased abundance of a microRNA targeting the NOXA mRNA. We demonstrate that miR-23a targets NOXA mRNA and that exposure to hyperthermia causes the depletion of miR-23a resulting in an increased abundance of NOXA mRNA and protein leading to cell death. Cells overexpressing HSP70 have elevated levels of miR-23a and its loss is less severe in heat-stressed cells. As a result, these cells accumulated less NOXA mRNA and protein and consequently resisted heat-induced apoptosis.

## Results

**Figure 1:**
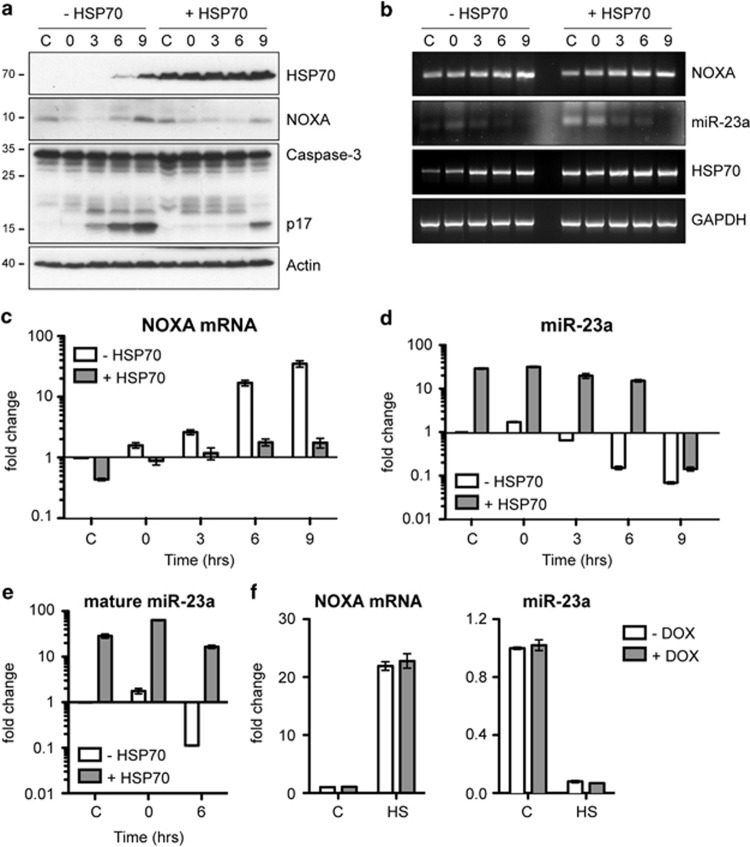
Exposure to hyperthermia results in increased NOXA protein and mRNA levels and is correlated with a decrease in the abundance of miR-23a. (**a**) Immunoblots of non-induced (- HSP70) and HSP70-expressing (+ HSP70) PErTA70 cells exposed to 43 °C for 1 h and then incubated at 37 °C for 0 to 9 h. Quantitation of the western blot data for *n*=4 repeats is shown in Supplementary Figure 1. (**b**) Semi-quantitative RT-PCR (quantification of *n*=3 repeats is shown in Supplementary Figure 2) and RT-qPCR analysis of (**c**) NOXA mRNA, (**d**) pri/pre-miR-23a and (**e**) mature miR-23a transcript levels in cells exposed to hyperthermia (mean±S.E.M, *n*=3). (**f**) As a control for the potential effects of doxycycline treatment on transcript levels, RT-qPCR was also performed using PErTA cells (the parental cell line that expresses the reverse tetracycline-controlled transactivator protein rtTA) that were either maintained at 37 °C or exposed to 43 °C for 1 h and then incubated at 37 °C for 9 h (HS)

We examined the expression of NOXA protein by western blotting in an acute lymphoblastic T-cell line (PEER) with tetracycline-regulated expression of HSP70 (PErTA70). As described previously,^13^ NOXA protein levels are initially reduced in non-induced cells exposed to hyperthermia (43 °C for 1 h), most likely due to the inhibition of transcription and translation in the heat-stressed cells and the short half-life of the protein. However, these levels increase when the cells are incubated at 37 °C reaching a level 2.4-fold greater than that of the control cells (Figure 1a and Supplementary Figure 1). The increase in NOXA protein levels corresponds to the time that the cells undergo apoptosis.^13, 16^ In cells overexpressing HSP70, the level of NOXA that was regained during incubation at 37 °C did not exceed the basal level and these cells had much less caspase-3 cleavage (Figure 1a and Supplementary Figure 1) as well as other indicators of apoptosis.^13, 16^

We next performed semi-quantitative RT-PCR to determine whether the accumulation of NOXA protein was due to increased mRNA expression (Figure 1b). Cells that were not induced to express HSP70 appeared to accumulate more NOXA mRNA than did the HSP70-expressing cells. Quantification of these results revealed a 2.5-fold increase in NOXA mRNA levels in the non-induced cells compared with a 1.4-fold increase in the HSP70-expressing cells (Supplementary Figure 2). We next used the more quantitative RT-qPCR technique to more accurately gauge the changes in NOXA mRNA expression (Figure 1c). Levels of NOXA mRNA increased approximately 30-fold in the non-induced cells but only about 2-fold in the HSP70-expressing cells. We considered the possibility that the increased levels of NOXA in heat-shocked cells could be due to the loss of an miRNA targeting the NOXA mRNA 3'UTR. A potential binding site for miR-23a was identified within the NOXA 3′ UTR sequence (position 100-107 relative to the stop codon) using the miRNA-target prediction database Targetscan. Analysis of miR-23a expression by RT-PCR and RT-qPCR revealed that levels were inversely correlated with that of NOXA mRNA in the heat-shocked cells (Figures 1b and d). Surprisingly, HSP70-expressing cells had elevated levels of miR-23a and maintained higher levels after heat shock exposure than did the non-expressing cells.

As the RT-qPCR analysis shown in Figure 1d measured levels of the pri- and pre-miR-23a transcripts, we also examined whether a similar pattern was observed for the mature miR-23a (Figure 1e). The relative levels of mature miR-23a were nearly identical to that of the pri/pre-miR-23a transcripts. The percentage of mature relative to total (pri/pre+mature) was approximately 36% for both the non-induced and the HSP70-expressing cells prior to heat shock treatment. By 6 h after heat shock, the percentage of mature transcripts was reduced to 26% in the non-induced cells but was not changed in the cells expressing HSP70. This suggests that hyperthermia has a modest affect on the maturation of miR-23a, but a much more significant impact on its level of expression.

To rule out the possibility that the changes in NOXA and miR-23a expression observed in the induced PErTA70 cells were mediated by doxycycline and not HSP70, we treated the parental cell line PErTA with doxycycline for 24 h and measured NOXA mRNA and miR-23a transcript levels by RT-qPCR in control and heat-shocked cells (Figure 1f and Supplementary Figure 3). Heat shock treatment increased NOXA mRNA and decreased miR-23a levels in a manner similar to what was observed in the non-induced PErTA70 cells. This pattern was not altered by doxycycline treatment indicating that the effects shown for the induced PErTA70 cells are attributable to HSP70.

**Figure 2:**
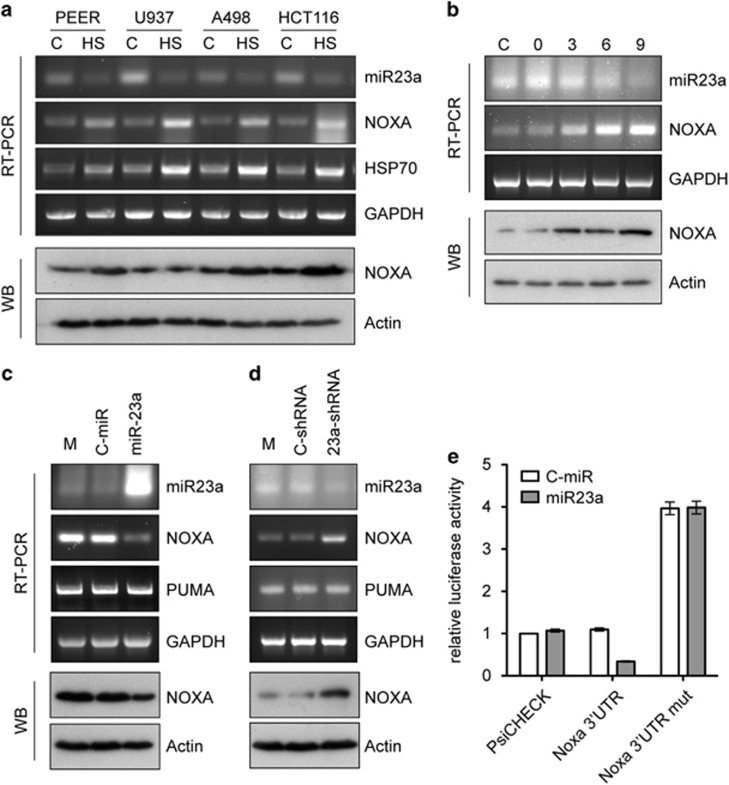
NOXA mRNA abundance is regulated by miR-23a binding to the NOXA mRNA 3′UTR. (**a**) Semi-quantitative RT-PCR analysis of pri/pre-miR-23a and NOXA mRNA transcript levels and western blot (WB) analysis of NOXA protein levels in response to hyperthermic exposure in a panel of human cancer cell lines: PEER (acute lymphoblastic leukemia), U937 (histiocytic lymphoma), A498 (kidney carcinoma) and HCT116 (colon carcinoma). Cells were exposed to 43 °C for 1 h and then incubated at 37 °C for 6 h. (**b**) RT-PCR analysis of pri/pre-miR-23a and NOXA transcript levels in HeLa cells exposed to 43 °C for 1 h and then incubated at 37 °C for 0 to 9 h. Levels of NOXA protein are also shown. (**c**) RT-PCR analysis of HeLa cells transiently transfected with either a control miRNA or miR-23a expression plasmid. (M=mock-transfected cells) (**d**) RT-PCR analysis of HeLa cells transiently transfected with a control shRNA or an shRNA targeting miR-23a. (**e**) Luciferase activity assay of HeLa cells transiently transfected with either a control luciferase expression plasmid (psiCHECK-2) containing a minimal 3′UTR with an SV40 polyA sequence, or a plasmid in which this sequence is replaced with the NOXA mRNA 3′UTR or the NOXA 3′UTR containing a mutation in the miR-23a binding site. Cells were co-transfected with the reporter plasmids plus either a control miRNA or an miR-23a expression plasmid (mean±S.E.M., *n*=3)

We next used semi-quantitative RT-PCR to determine whether a similar pattern of miR-23a and NOXA mRNA expression occurred in other cells exposed to hyperthermia (Figure 2a). Although basal and heat-induced levels of expression varied for each of the human cancer cell lines, in every case, heat shock treatment resulted in an increase in NOXA mRNA and a corresponding decrease in miR-23a levels. The increased abundance of NOXA mRNA was accompanied by an increase in NOXA protein levels in each of the heat-shocked cells with the exception of U937. We also examined the temporal changes in NOXA mRNA and miR-23a levels in heat-stressed HeLa cells (Figure 2b). Again, an inverse correlation between levels of NOXA mRNA and miR-23a was observed.

To directly determine whether miR-23a regulated NOXA mRNA levels, we transiently transfected HeLa cells with a miR-23a expression vector or a non-targeting (C-miR) vector and performed RT-PCR (Figure 2c). Overexpression of miR-23a substantially reduced the abundance of NOXA mRNA and protein but not Puma mRNA, which does not contain a miR-23a-binding site within its 3′UTR. Additionally, we transiently transfected HeLa cells with a plasmid encoding an shRNA targeting the miR-23a sequence and found that this resulted in an increase in NOXA mRNA and protein abundance (Figure 2d). Transfection with a mutant version of this plasmid in which five nucleotides in the miR-23a targeting sequence were altered had no effect on the levels of miR-23a or NOXA mRNA and protein. Finally, we cloned the NOXA 3′UTR sequence from human genomic DNA and inserted it into a luciferase reporter plasmid for transient co-transfection assays with the miR-23a expression plasmid or C-miR. We also created a mutated version of the NOXA 3'UTR reporter plasmid in which the miR-23a-binding site was altered by site-directed mutagenesis. In transiently transfected HeLa cells, we observed a 65% reduction in luciferase activity from the NOXA 3′UTR reporter plasmid when miR-23a was overexpressed (Figure 2e). Also, deletion of the miR-23a-binding site from the NOXA 3'UTR resulted in a fourfold increase in luciferase activity that was unaffected by miR-23a expression. Together, these data demonstrate that miR-23a is capable of binding the NOXA mRNA 3′UTR resulting in a reduction in NOXA mRNA levels.

**Figure 3:**
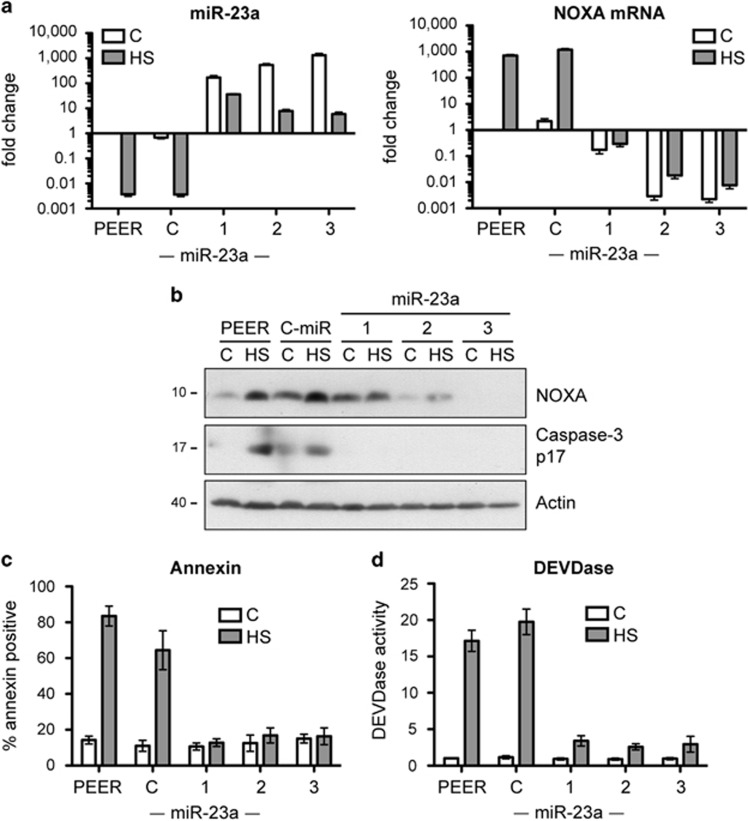
Overexpression of miR-23a in stably transfected PEER cells results in reduced NOXA mRNA and protein levels and increased resistance to heat-induced apoptosis. (**a**) RT-qPCR analysis of miR-23a and NOXA mRNA levels in the parental PEER cell line, a stable clone expressing a control miRNA (**c**) and in three stable clones expressing miR-23a (mean±S.E.M., *n*=3). miR-23a transcript levels represent both pri-miRNA and pre-miRNA. (**b**) Immunoblot analysis of NOXA and cleaved caspase-3 levels in cells exposed to heat shock. (**c**) Quantification of apoptosis by Annexin-V staining and flow cytometry (mean±S.E.M., *n*=3). (**d**) Caspase-3 activity assay (mean±S.E.M., *n*=3). Heat-shocked cells were exposed to 43 °C for 1 h and then incubated at 37 °C for 6 h (HS)

In order to assess the consequences of miR-23a expression on resistance to heat-induced apoptosis, we created stably transfected cell lines overexpressing miR-23a using the same parental cell line (PEER) that was used to create the PErTA70 cell line. Analysis of miR-23a levels by RT-qPCR showed a 100 to over 1000-fold elevation in miR-23a levels in the three overexpressing clones (Figure 3a). These cells had correspondingly decreased levels of NOXA mRNA. Exposure to hyperthermia reduced miR-23a levels in all clones; however, the overexpressing clones still maintained levels that were higher than the basal level in the parental cell line. As a result, while NOXA mRNA levels increased nearly 1000-fold in the parental and control miRNA-expressing cell line, NOXA mRNA levels in the miR-23a-expressing clones remained repressed. As a consequence, basal and heat-induced levels of NOXA protein were also repressed in these cells and heat shock treatment failed to result in caspase-3 processing (Figure 3b). The extent of apoptosis was quantitated in these cells by measuring Annexin-V staining (Figure 3c) and caspase-3 activity (Figure 3d). While heat shock treatment resulted in a significant increase in both indicators of apoptosis in the parental and control miRNA lines, the miR-23a-expressing clones were essentially unaffected by the heat treatment. Therefore, forced overexpression of miR-23a reduces NOXA mRNA and protein levels and protects cells from heat-induced apoptosis.

**Figure 4:**
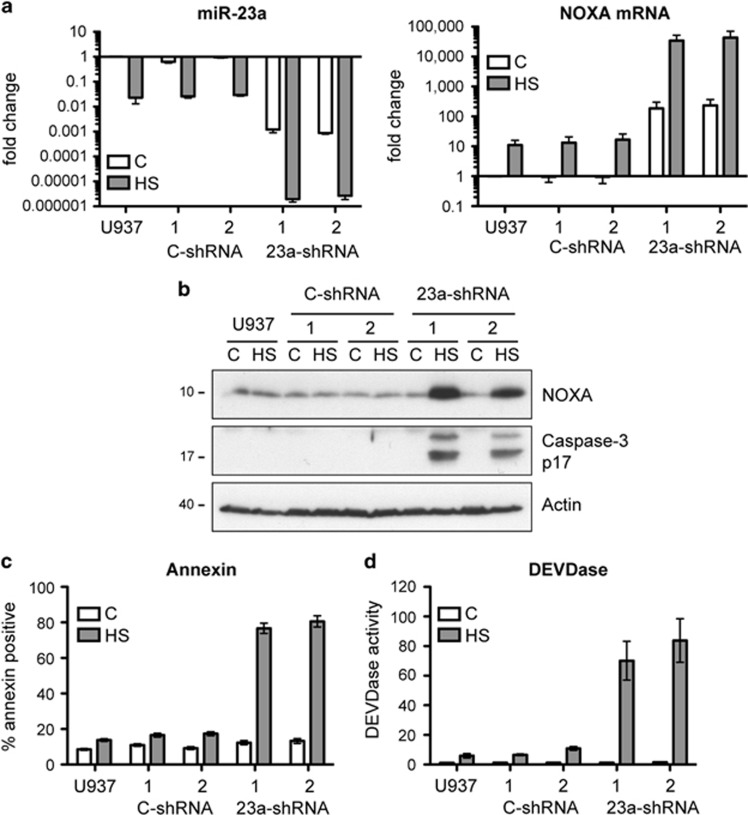
Overexpression of an shRNA targeting miR-23a in stably transfected U937 cells results in increased NOXA mRNA and protein levels and increased sensitivity to heat-induced apoptosis. (**a**) RT-qPCR analysis of miR-23a and NOXA mRNA levels in the parental U937 cell line, two stable clones expressing a control shRNA and two stable clones expressing a miR-23a targeting shRNA (mean±S.E.M., *n*=3). miR-23a transcript levels represent both pri-miRNA and pre-miRNA. (**b**) Immunoblot analysis of NOXA and cleaved caspase-3 levels in heat-shocked cells. (**c**) Quantification of apoptosis by Annexin-V staining and flow cytometry (mean±S.E.M., *n*=3). (**d**) Caspase-3 activity assay (mean±S.E.M., *n*=3). Heat-shocked cells were exposed to 43 °C for 1 h and then incubated at 37 °C for 6 h (HS)

To further substantiate a role for miR-23a in NOXA mRNA regulation and heat resistance, we knocked down the expression of miR-23a in stably transfected U937 cells using an shRNA targeting miR-23a. U937 cells were chosen because they have higher basal levels of miR-23a compared with the non-induced PErTA70 cells (Figure 2a) and are more resistant to hyperthermia than either the non-induced PErTA70 cells or the parental PEER cell line (Supplementary Figure 4). miR-23a and NOXA mRNA levels were examined by RT-qPCR in the parental U937 cell line, in two clones expressing the miR-23a targeting shRNA (23a-shRNA) as well as two clones (C-shRNA) expressing a non-targeting shRNA (Figure 4a). Levels of miR-23a were reduced about 40-fold in the parental and non-targeting shRNA clones following exposure to hyperthermia. However, miR-23a levels were 1000-fold less in the miR-23a shRNA-expressing clones relative to the parental line, and exposure to hyperthermia further reduced these levels by an additional 500-fold. NOXA mRNA levels in the miR-23a shRNA cells were elevated by approximately 200-fold in non-stressed cells relative to the parental cells and exposure to hyperthermia resulted in a further 200-fold increase relative to their own basal level. Western blot analysis revealed that unlike the PEER cell line, exposure of U937 cells to 43 °C hyperthermia did not significantly affect NOXA protein levels or result in caspase-3 processing (Figure 4b). However, NOXA protein levels and caspase-3 processing were substantially enhanced when the miR-23a shRNA-expressing cells were exposed to hyperthermia. Furthermore, these cells were highly sensitized to heat-induced apoptosis as shown by an increased percentage of Annexin-V staining (Figure 4c) and DEVDase activity (Figure 4d). Therefore, knockdown of miR-23a increases NOXA mRNA levels and sensitizes cells to heat-induced apoptosis.

**Figure 5:**
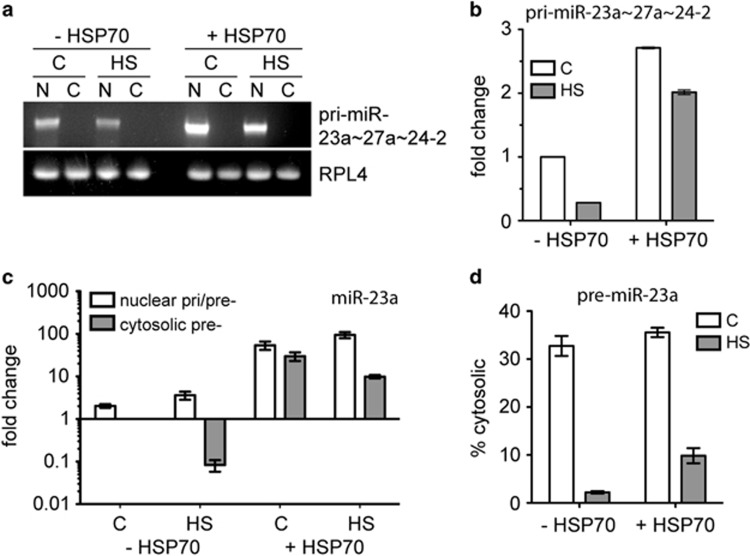
Overexpression of HSP70 enhances the transcription and nuclear export of miR-23a. (**a**) Non-induced and induced (HSP70-expressing) PErTA70 cells were exposed to 43 °C for 60 min and then incubated at 37 °C for 6 h at which time they were lysed and separated by centrifugation into nuclear (N) and cytosolic (C) fractions. Semi-quantitative RT-PCR was performed to measure relative levels of the pri-miR-23a~27a~24-2 cluster. Shown is a representative result. (**b**) Quantification of the results shown in (**a**) (mean±S.E.M., *n*=3). (**c**) RT-qPCR analysis of pri- and pre-miR-23a levels in the isolated fractions (mean±S.E.M., *n*=3). Values are expressed relative to the cytosolic value for non-induced control cells. (**d**) Comparison of the percent cytosolic pre-miR-23a from the results shown in panel (**c**) (mean±S.E.M., *n*=3)

Having established that miR-23a regulates NOXA expression and that this is a critical factor regulating heat resistance, we next sought to determine the mechanism responsible for the elevated levels of miR-23a in cells overexpressing HSP70. We considered the possibility that HSP70 could affect miR-23a transcription, nuclear export or turnover. miR-23a is transcribed by RNA polymerase II as part of a pri-miRNA cluster that includes miR-27a and miR24-2.^17^ We measured the levels of this pri-miRNA by semi-quantitative RT-PCR in the PErTA70 cell line (Figure 5a). Expression of the pri-miRNA transcript was 2.7-fold higher in the HSP70-expressing cells compared with the non-induced cells indicating either elevated rates of transcription or enhanced stability (Figures 5a and b). Levels of the pri-miRNA were reduced following exposure to hyperthermia in both the non-induced and the HSP70-expressing cells, although the cells with HSP70 still contained twofold higher levels of the pri-miRNA after heat shock than did the non-induced cells that were not exposed to hyperthermia (Figures 5a and b). Levels of the pri-miRNA were reduced by only 26% in the HSP70-expressing cells but by over 72% in the non-induced cells.

To address differences in pre-miRNA nuclear export, we measured pri/pre-miR-23a levels by RT-qPCR in isolated nuclear and cytosolic fractions (Figure 5c). Fold changes in levels are expressed relative to the cytosolic value of the control non-induced cells. Cytosolic levels of pre-miR-23a decreased in both the non-induced and HSP70-expressing cells; however, the HSP70-expressing cells maintained higher levels of cytosolic pre-miR-23a as a result of its higher total abundance. Calculation of the percentage of total pre-miR-23a that was present in the cytosolic fraction shows that HSP70 had a beneficial effect on the nuclear export of pre-miR-23a (Figure 5d). Cytosolic levels of pre-miR-23a dropped 15-fold in the non-induced cells but only 3.6-fold in the HSP70-expressing cells.

**Figure 6:**
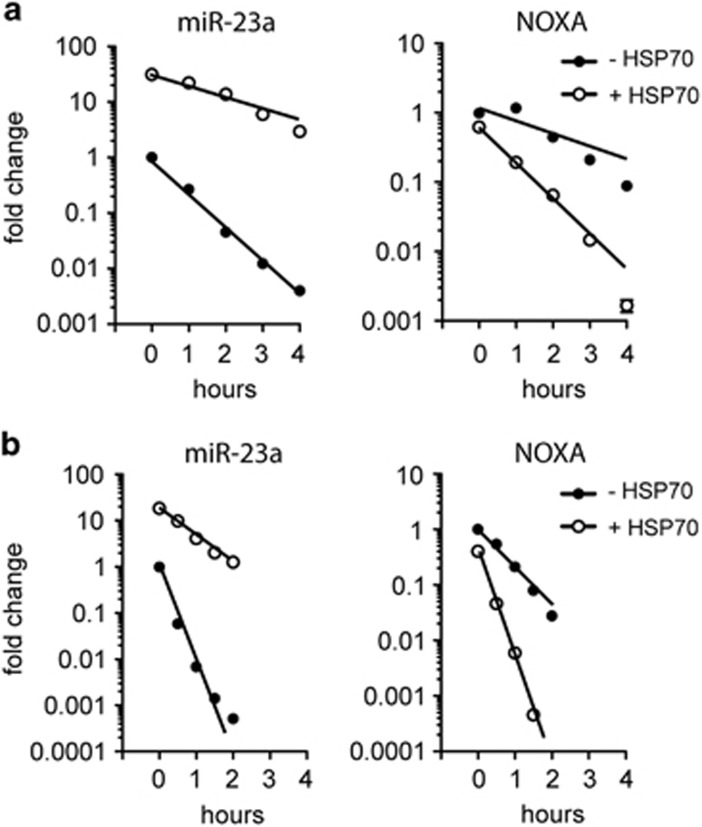
Overexpression of HSP70 enhances the stability of miR-23a RT-qPCR analysis of pri/pre-miR-23a and NOXA mRNA levels in non-induced (-HSP70) and induced (+HSP70) PErTA70 cells that were (**a**) incubated with actinomycin D for 0 to 4 h at 37 °C or (**b**) exposed to 43 °C for 1 h before incubation with actinomycin D for 0 to 2 h at 37 °C (mean±S.E.M., *n*=3)

To determine whether the elevated levels of miR-23a in the HSP70-expressing cells is due to a reduced rate of turnover, we incubated cells with actinomycin D and measured the remaining amount of miR-23a at various times during incubation at 37 °C (Figure 6a). In the non-induced cells, miR-23a levels were reduced 10-fold within 90 min, whereas in the HSP70-expressing cells this required nearly 4 h representing a 2.7-fold slower turnover rate. Consequently, NOXA mRNA levels decayed more rapidly in the HSP70-expressing cells compared with the non-induced cells (Figure 6a). We also examined the effect of exposure to hyperthermia on miR23a stability. For this, we exposed cells to 43 °C for 60 min and then incubated them at 37 °C in the presence of actinomycin D for 30–120 min (Figure 6b). Heat shock treatment resulted in a more rapid turnover of both miR-23a and NOXA mRNA. In the non-induced cells, miR-23a levels decreased by more than 10-fold within 30 min. However, in the presence of HSP70 a 10-fold reduction did not occur until about 90 min. Therefore, the stability of miR-23a is enhanced by threefold in HSP70-expressing cells exposed to hyperthermia compared with the non-induced cells. The enhanced stability of miR-23a in the HSP70-expressing cells was again correlated with a more rapid turnover of NOXA mRNA following heat exposure.

In summary, miR-23a levels are reduced in cells exposed to hyperthermia most likely due to the combined effects of the inhibitory effects of high temperature on RNA polymerase II-directed transcription, an impairment of pre-miR-23a nuclear export and an elevated rate of turnover. HSP70-expressing cells have higher levels of miR-23a, which is primarily due to a reduced rate of turnover, and maintain high levels of miR-23a following exposure to hyperthermia in part due to the protective effects of HSP70 on pri-miRNA transcription, pre-miRNA nuclear export and maturation. However, the increased stability of miR-23a provided by HSP70 expression is most likely the major factor contributing to its preservation in heat-shocked cells leading to a more rapid loss of NOXA mRNA.

## Discussion

All living organisms must employ strategies to cope with environmental stress, particularly proteotoxic stress which can impair cell structure and function, and if uncontrolled, will lead to cell death.^18^ Although removal of irreparably damaged cells by apoptosis is beneficial to the organism, adaptive measures must act to prevent inappropriate cell elimination when faced with fluctuating environmental conditions. The heat shock response is an evolutionarily conserved response that allows cells to increase their level of resistance to proteotoxic stress by stress-dependent synthesis of heat shock proteins.^19^ The primary function of this family of molecular chaperones is the maintenance of proteostasis by assisting in the proper folding of cellular proteins.^20^ This ability provides a powerful anti-apoptotic function not only by preventing protein misfolding in general but also by intervening at key steps in apoptotic pathways. For example, exposure of cells to hyperthermia triggers BAX oligomerization, cytochrome c release and caspase activation all of which are inhibited in cells overexpressing the molecular chaperone HSP70.^11, 16, 21^ As NOXA protein levels increase following heat shock treatment, we explored the possibility that this was due to the loss of a microRNA targeting the NOXA mRNA. We have now demonstrated that NOXA expression is regulated by miR-23a, that synthesis of this microRNA is repressed in cells exposed to hyperthermia and that cells expressing HSP70 resist this repression. Consequently, HSP70 can protect cells from heat-induced apoptosis by maintaining miR-23a levels and thereby prevent the accumulation of NOXA mRNA and protein.

BH3-only proteins act at the critical tipping point in the cell death decision-making process by overcoming the inhibitory effect that the anti-apoptotic BCL2 proteins have on BAX/BAK activation.^3^ NOXA performs this task by selectively binding MCL1 and A1 and so must act together with other BH3-only proteins in order to target the remaining pool of anti-apoptotic BCL-2 family members. MCL1 is often overexpressed in malignant cells, particularly in leukemia where the NOXA/MCL1 ratio determines sensitivity to chemotherapy and BH3-memitic drugs.^22^ NOXA expression can be increased transcriptionally through p53-dependent and -independent mechanisms and also by regulating protein turnover.^6^ NOXA has a very short half-life (~30 min) and is degraded by the proteasome; however, its turnover is not entirely dependent upon ubiquitination.^8^ The rate of NOXA protein turnover could be affected by hyperthermia and influenced by the presence of HSP70. Our results show that the abundance of NOXA is controlled by microRNA regulation, which provides a rapid mechanism to increase NOXA protein levels in cells exposed to proteotoxic stress.

The critical role that microRNAs play in the regulation of stress responses has only recently gained significant attention.^23^ Roles in apoptosis and tumorigenesis are well-documented ^24, 25^ and not surprisingly many apoptotic regulators including BCL2 family members and caspases are regulated by microRNAs that in many cases have altered patterns of expression in cancer. MiR-23a is transcribed as part of the miR-23a~27a~24-2 cluster, which is deregulated in several diseases including cancer where expression is elevated.^17^ This cluster was found to be amplified in a mouse model of colon cancer and the elevated miR-23a expression was shown to promote the transition of indolent adenomas to invasive colorectal cancers.^26^ Elevated expression of miR-23a was also documented in human colorectal cancers. MiR-23a regulates APAF1^27^ and caspase-7^28^ and together with our finding that it also targets NOXA reveals that this microRNA coordinately antagonizes the expression of multiple apoptotic regulators and therefore its downregulation during proteotoxic stress ensures effective apoptotic pathway activation.

There are only a few reports on microRNA expression in heat-stressed cells.^29^ In general, the expression of some microRNAs were found to be enhanced whereas others were reduced.^30, 31, 32, 33^ None of these studies examined whether HSP70 expression could modulate the heat-induced changes in miRNA expression patterns. HSC70 and HSP90 are known to assist in the loading of miRNA duplexes into the RISC complex by an ATP-dependent conformational change of the Ago proteins.^34^ Our results point to a role for HSP70 in regulating the cellular abundance of microRNAs. Heat shock is a well-documented inhibitor of transcription and translation, however, both of these processes recover more rapidly in cells overexpressing HSP70.^35^ As the majority of miRNA transcripts are derived from RNA polymerase II-directed transcription, including miR-23a,^36^ we suggest that hyperthermia could cause a general reduction in the abundance of microRNAs and that this could have a significant impact on the abundance of proteins that are short-lived such as NOXA. The protective effect that HSP70 has on the recovery of transcription and translation following heat stress would help to buffer these changes and could significantly influence cell fate. Hyperthermia is also known to disrupt mRNA precursor processing and prior heat shock protein synthesis protects intron splicing in heat-stressed cells.^37^ We speculate that pri-microRNA processing is another activity that could be disrupted in heat-stressed cells and which could be protected by HSP70.

Tumor cells often have elevated levels of heat shock proteins including HSP70, which provide a selective pro-survival advantage that contributes to the process of tumorigenesis.^1^ HSP70 has been implicated in the suppression of multiple apoptotic regulators from stress kinase signaling to BCL2 and caspase family regulation. Our results demonstrate that HSP70 can also intervene in pathways leading to stress-induced cell death by altering the microRNA profile of cells. The elevated levels of miR-23a in HSP70-expressing cells could result from inhibition of microRNA degradation perhaps by preventing its deadenylation in the nucleus and/or by stabilizing interactions with Drosha/DGCR8 or Exportin-5. Alternatively, HSP70 could affect the abundance or activity of transcription factors that are responsible for the regulation of miR-23a expression. We suspect that alteration of microRNA expression could be a general mechanism by which HSP70 contributes to tumorigenesis.

## Materials and Methods

### Cell lines and treatments

The PErTA70 cells with tetracycline-regulated expression of HSP70 (HSPA1) were described previously^16^ and are derived from the acute lymphoblastic T-cell line PEER that was engineered to express the reverse tetracycline-controlled transactivator protein rtTA (PErTA). A control miR expression plasmid and an miR-23a overexpression plasmid containing the pre-miR-23a sequence in the 3′UTR of a GFP reporter plasmid (pEZX-MR04) were obtained from GeneCopoeia (Rockville, MD, USA). PEER cells stably overexpressing the control miR or miR-23a were generated by electroporation and selection with puromycin at 0.8 *μ*g/ml (HyClone Thermo Scientific, Markam, ON, Canada). Drug-resistant clones were screened by flow cytometry using a Beckman Coulter FC500 (Beckman Coulter Canada, Mississauga, ON, Canada). An shRNA targeting miR-23a (5′-ggaaatccctggcaatgtgat-3′) was constructed using pSUPER-puro (Oligoengine, Seattle, WA, USA). A control shRNA plasmid was created by altering five nucleotides in the miR-23a targeting sequence (5′-gtacatccatggcactgtggt-3′). U937 cells stably expressing these plasmids were generated by electroporation and selection with puromycin at 0.025 *μ*g/ml. Drug-resistant clones were screened by PCR analysis of genomic DNA. Transient transfection of HeLa cells was performed using calcium phosphate precipitation.

For heat shock treatments, suspension cells (PEER, U937) were pelleted and resuspended in fresh media (RPMI with 10% FBS and 10 mM HEPES pH 7.2; all media supplies were obtained from HyClone) at a concentration of 5 × 10^6^ cells/ml in a 15 ml centrifuge tube and submerged in a circulating water bath at 43 °C for 1 h. The cells were either collected immediately after the heat shock treatment or transferred to cell culture flasks with fresh media to dilute the cells to 1 × 10^6^/ml and incubated at 37 °C for up to 9 h. Adherent cells (HeLa, A498, HCT116) were grown in DMEM with 10% FBS and heated while attached to 10 cm culture dishes (with the addition of 10 mM HEPES pH7.2) that were wrapped in parafilm and floated on top of the water surface of a circulating water bath.

Nuclear export of miR-23a was examined by RT-qPCR in detergent lysed cells. Following treatment, cells were collected by centrifugation, washed twice with cold PBS and resuspended in 500 *μ*l of a hypotonic lysis buffer (20 mM Tris-HCl, pH 7.5, 10 mM NaCl, 3 mM MgCl_2_). After incubation on ice for 15 min, 25 *μ*l 10% NP40 was added and the samples were vortexed at high speed for 10 s. The lysates were separated into a nuclear pellet fraction and a cytosolic supernatant fraction by centrifugation at 3000 r.p.m. for 10 min at 4 ºC. The pellet fraction was washed by resuspension in 500 *μ*l hypotonic lysis buffer and centrifuged again. RNA was then isolated from each fraction.

To measure RNA stability, cells were pelleted and resuspended in fresh media (RPMI with 10% FBS and 10 mM HEPES pH 7.2) at a concentration of 1 × 10^6^ cells/ml. Actinomycin D (Sigma-Aldrich, Oakville, ON, Canada) was added to a final concentration of 1 *μ*M and the cells were incubated at 37 °C for up to 4 h. Cells were collected each hour (or every 30 min for heat-shocked cells) by centrifugation, washed with PBS, and RNA was isolated.

### RT-PCR and RT-qPCR

Cells were collected by centrifugation, washed with PBS, and RNA was isolated using TRIzol Reagent (Invitrogen-Life Technologies, Burlington, ON, Canada). RNA was quantified by Nanodrop and cDNA was synthesized from 5 μg of RNA using an oligo(dT) primer and SuperScript II Reverse Transcriptase kit in a total volume of 19 *μ*l (Invitrogen-Life Technologies). For the miR-23a results shown in Figure 1b, RNA was first treated with poly(A) polymerase (New England Biolabs, Pickering, ON, Canada) before reverse transcription and PCR analysis using primers: miR-23a-fwd: 5′-ccgccgggatccacggccggctggggttcc-3′ miR-23a-rev: 5′-ccgccgaagcttcagagctcagggtcggttgg-3′. For the detection of pri-miR-23a~27a~24-2 (Figure 5a), RNA was reverse-transcribed with the gene-specific primer: 5′-aaccccacccaccacatccctcctccagac-3′, followed by PCR using primers: pri-fwd: 5′-ccctgttcctgctgaactgagccagtgtac-3′, pri-rev: 5′- cgcccggtgcccccctcacccctgtgccac-3′. PCR was carried out using GOTaq Flexi DNA Polymerase (Promega, Madison, WI, USA) with the following gene-specific primers: NOXA-fwd: 5′-tgttcgtgttcagctcgcgt-3′ NOXA-rev: 5′-agcacactcgacttccagc-3′ miR-23a-fwd: 5′-ctggggttcctggggatg-3′ miR-23a-rev: 5′-ggtcggttggaaatccctg-3′ PUMA-fwd: 5′-gacctcaacgcacagta-3′ PUMA-rev: 5′-ctaattgggctccatct-3′ HSP70-fwd: 5′-ttccgtttccagcccccaatc-3′ HSP70-rev: 5′-cgttgagccccgcgatgaca-3′ GAPDH-fwd: 5′-cccctggccaaggtcatccatgacaacttt-3′ GAPDH-rev: 5′-ggccatgaggtccaccaccctgttgctgta-3′. Each 25 *μ*l reaction contained 10 *μ*M primers and 1 *μ*l cDNA in 1 × GOTaq Flexi buffer. All PCR reactions were 30 cycles except for miR-23a, which was 35 cycles. PCR products were mixed with RedSafe dye (FroggaBio, Toronto, ON, Canada) analyzed by agarose gel electrophoresis and imaged using a Bio-Rad ChemiDoc XRS^+^ imaging system (Bio-Rad, Laboratories, Mississauga, ON, Canada).

For RT-qPCR, cDNA was synthesized from 0.017 *μ*g purified RNA and random primers using the High Capacity cDNA Reverse Transcription kit (Applied Biosystems-Life Technologies, Burlington, ON, Canada). qPCR was performed using PerfeCTa FastMix II from Quanta Biosciences and the Applied Biosystems StepOnePlus real-time PCR instrument at the University of Guelph Advanced Analysis Centre. Primer-specific amplification efficiencies were determined by constructing a standard curve of serially diluted cDNA. For each sample, the relative amount of starting template was determined by calculating the ΔΔCt after correcting the Ct values for expression of RPL4. Primers used for qPCR were: miR-23a-fwd: 5′-ctggggttcctggggatg-3′ miR-23a-rev: 5′-ggtcggttggaaatccctg-3′ NOXA-fwd: 5′-gctggaagtcgagtgtgcta-3′ NOXA-rev: 5′-ggagtcccctcatgcaagtt-3′ RPL4-fwd: 5′-gctctggccagggtgcttttg-3' RPL4-rev: 5′-atggcgtatcgtttttgggttgt-3′. Note that these miR-23a primers will detect both pri- and pre-miR-23a. To measure the relative abundance of mature miR-23a, we used a stem-loop RT-qPCR strategy as described by Kramer.^38^ The RT reaction was performed using the stem-loop primer 5'-gtcgtatccagtgcagggtccgaggtattcgcactggatacgacggaaat-3'. Primers for qPCR were: mature miR-23a-fwd: 5′-gagcgggatcacattgcca-3′ mature miR-23a-rev: 5′-ccagtgcagggtccgaggta-3′.

### Immunoblotting

Control and heat-shocked cells were collected, lysed and immunoblotting performed as described previously.^11^ The following antibodies were used for immunoblotting: Actin (ACTN05: NeoMarkers, Fremont, CA, USA), Caspase-3 (BML-SA320: Enzo Life Sciences, Farmingdale, NY, USA), HSP70 (C92F3A-5: Enzo), NOXA (114C307.1: Enzo). Following exposure of the blots to film, the images were scanned and analyzed using Image J software (Research Services Branch, National Institute of Mental Health, USA).

### Luciferase reporter assays

Reporter plasmids were created using the psiCHECK-2 plasmid (Promega) into which was cloned the NOXA mRNA 3'UTR from genomic DNA using primers: fwd: 5′-gactagctcgagtgactgcatcaaaaacttgcatgagg-3′, rev: 5′-cacagtgcggccgcaattaaagtgtaagtcccttgagag-3′ (the complimentary genomic sequence is underlined) downstream of the Renilla luciferase coding sequence. A mutant version was constructed in which the miR-23a-binding site was altered by site-directed mutagenesis replacing the sequence 5′-aatgtgaa-3′ with a BstEII site 5′-tccactgg-3′. HeLa cells were transiently transfected by calcium phosphate precipitation using 5 ng of reporter plasmid and 10 *μ*g of either the GeneCopoeia miR-23a overexpression plasmid or the control miRNA plasmid. Cells were harvested 20 h after transfection and lysed in 60 *μ*l of passive lysis buffer. Renilla and firefly luciferase activity was measured with the dual-luciferase reporter assay system (Promega) in a Turner Instruments luminometer (Sunnyvale, CA, USA). Renilla values were standardized to the firefly activity to account for transfection efficiency.

### Apoptosis measurements

Annexin-V staining was performed using a Beckman Coulter FC500 flow cytometer on cells stained with Annexin-V, Alexa Fluor 647 Conjugate (Life Technologies). Caspase-3 activity assays were performed by measuring the cleavage of DEVD-AMC (Enzo Life Sciences) in cell extracts using a BioTek fluorescence plate reader (Winooski, VT, USA) as described previously.^11^ The relative fluorescence units of AMC released per minute per microgram of protein were calculated for each sample and are plotted relative to the non-stressed parental cell line being set to a value of one.

## Abbreviations

RT-qPCR: reverse transcription-quantitative real-time polymerase chain reaction
UTR: untranslated region
pri/pre-miRNA: primary/precursor microRNA
shRNA: short-hairpin RNA
DEVDase: proteolytic activity cleaving after aspartic acid in the peptide DEVD
