# Supplementary figures and images for: The elimination of miR-23a in heat-stressed cells promotes NOXA-induced cell death and is prevented by HSP70
Source: Cell Death Dis. 2014 Nov 27;5(11):e1546–. doi: 10.1038/cddis.2014.484 (PMC4260742; doi:10.1038/cddis.2014.484)

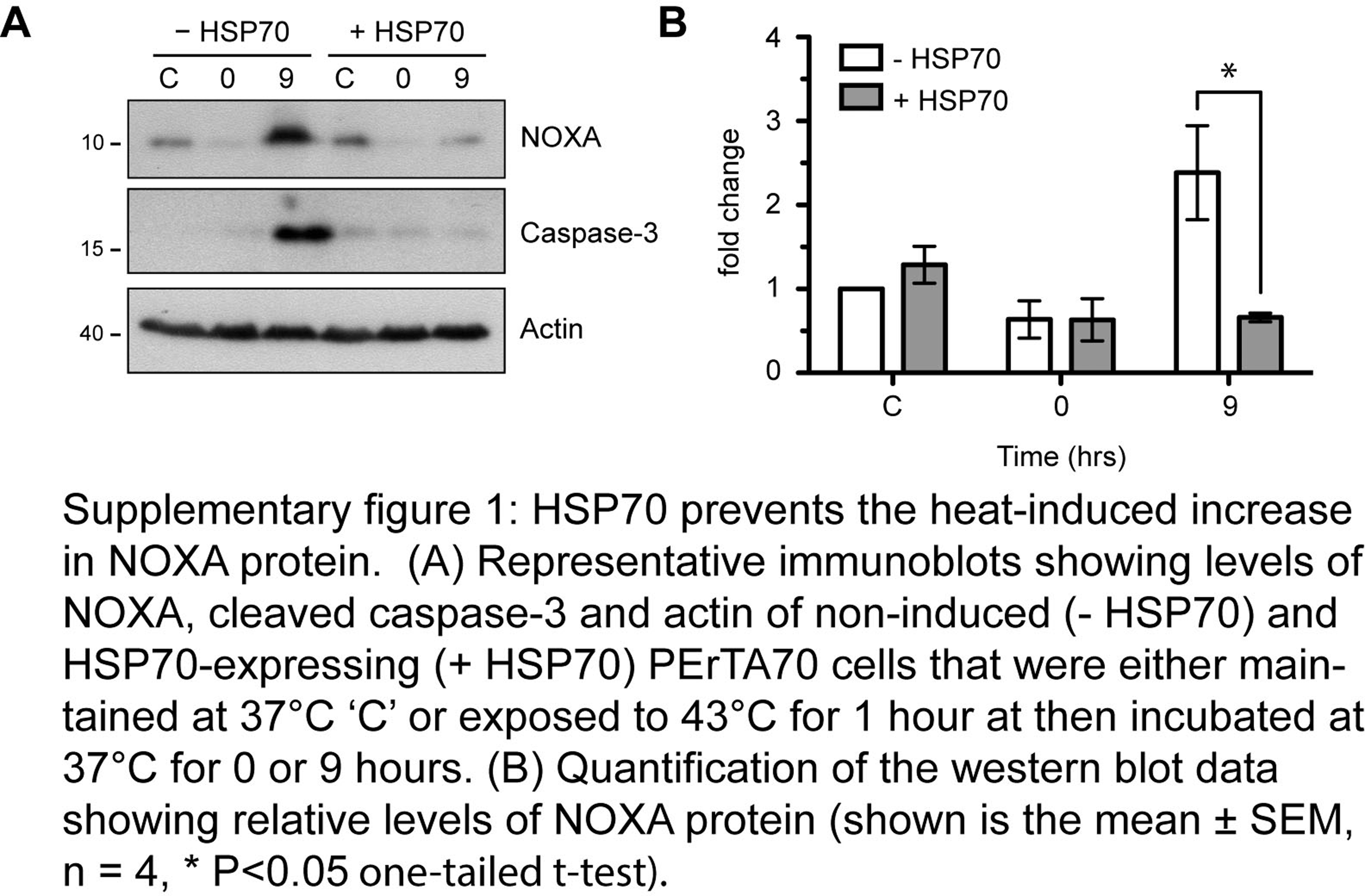

Supplement: Supplementary Figure 1 [file cddis2014484x1.tif]

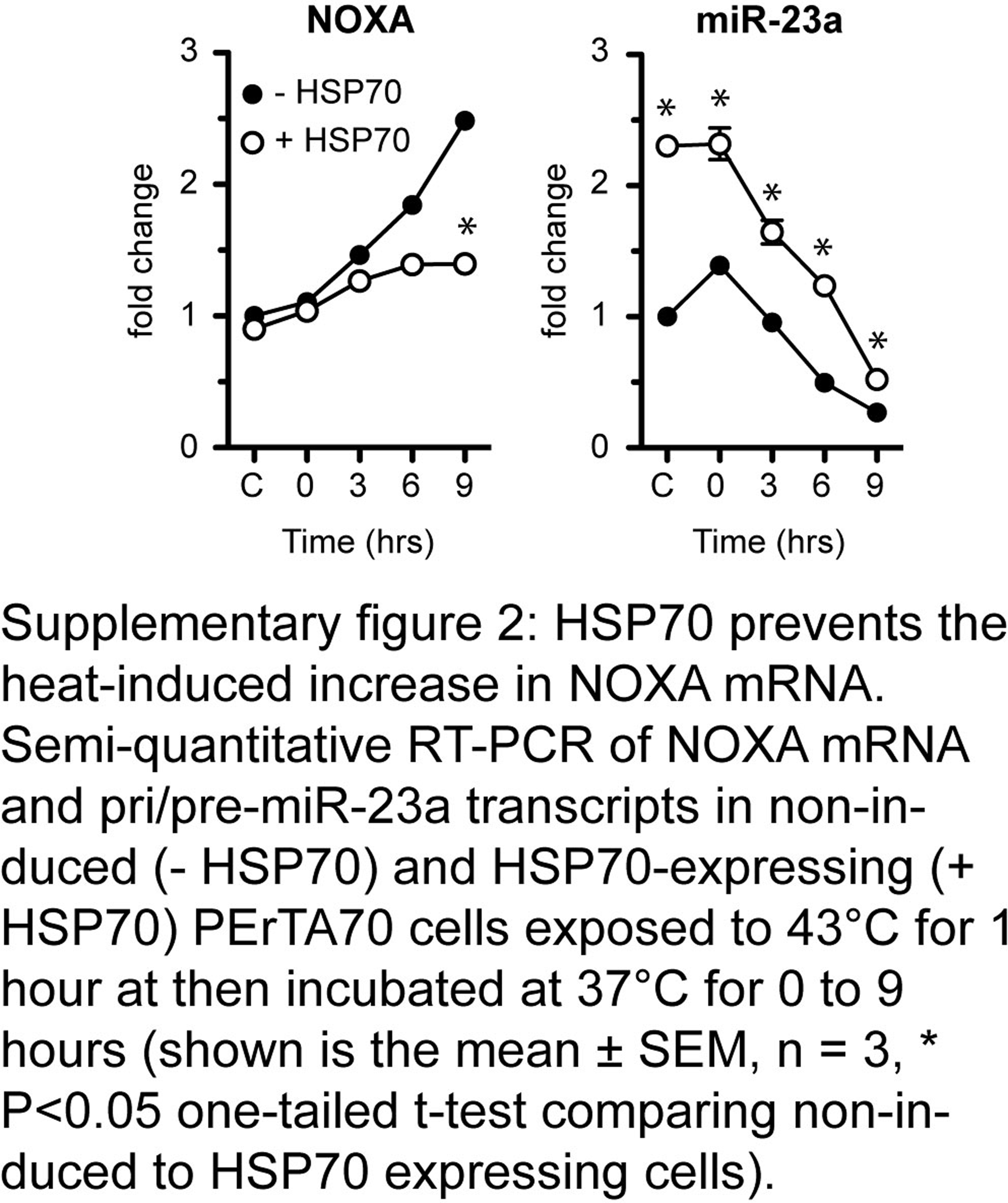

Supplement: Supplementary Figure 2 [file cddis2014484x2.tif]

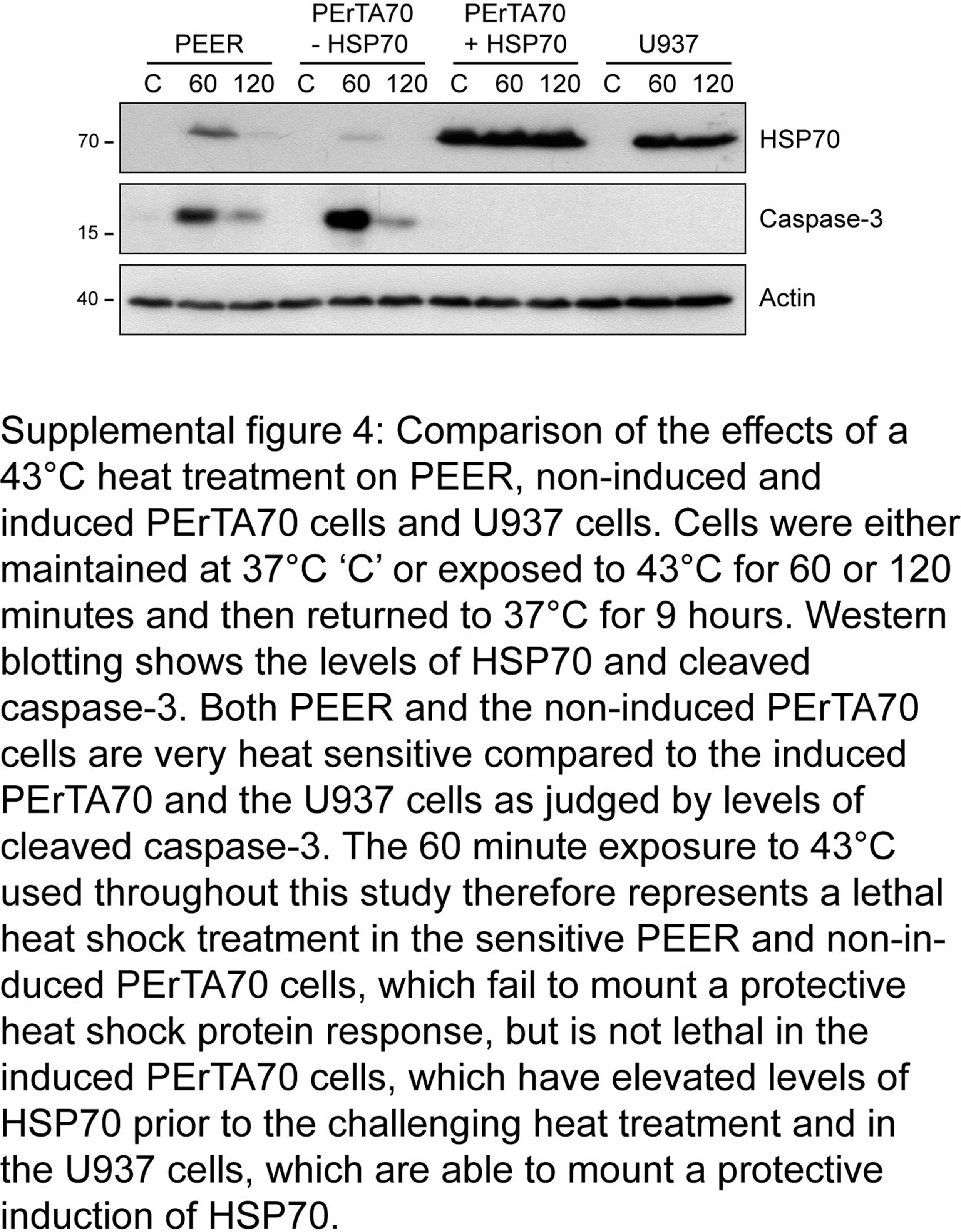

Supplement: Supplementary Figure 3 [file cddis2014484x3.tif]

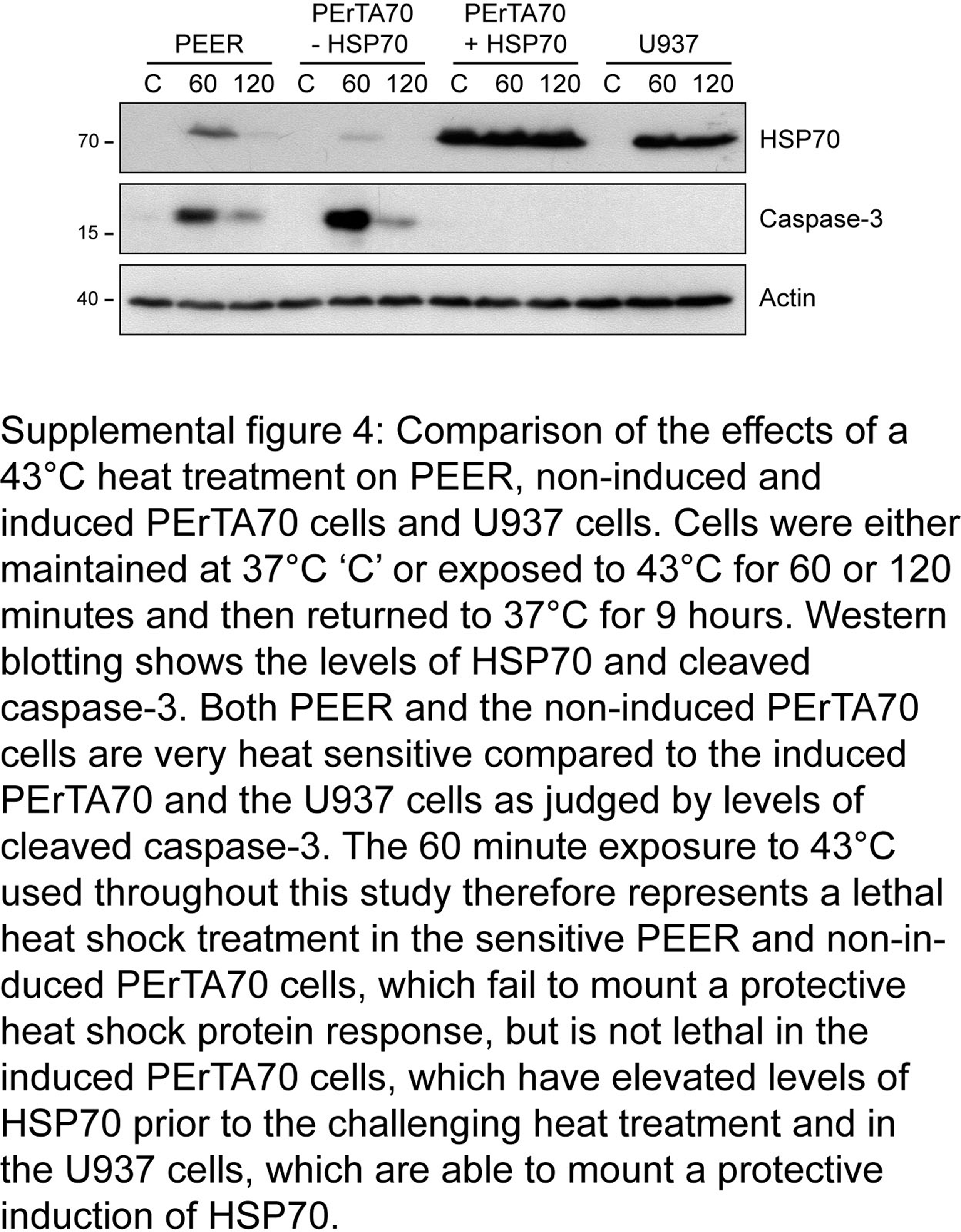

Supplement: Supplementary Figure 4 [file cddis2014484x4.tif]
